# Ursolic acid ameliorates cerebral ischemia-reperfusion injury by inhibiting NF-κB/NLRP3-mediated microglia pyroptosis and neuroinflammation

**DOI:** 10.3389/fphar.2025.1622131

**Published:** 2025-07-11

**Authors:** Junbin Liu, Xiaoting Zhang, Jingpei Guo, Yun Zhang, Jinming Fan, Junfeng Liu, Jiawen Chen, Jiawei Jiang, Benshuai Yu, Ke Zhang, Bin Zhou

**Affiliations:** ^1^ Center of Interventional Medicine, The Fifth Affiliated Hospital of Sun Yat-Sen University, Zhuhai, Guangdong, China; ^2^ Guangdong Provincial Key Laboratory of Biomedical Imaging and Guangdong Provincial Engineering Research Center of Molecular Imaging, The Fifth Affiliated Hospital of Sun Yat-Sen University, Zhuhai, Guangdong, China; ^3^ Guangdong-Hong Kong-Macao University Joint Laboratory of Interventional Medicine, The Fifth Affiliated Hospital, Sun Yat-Sen University, Zhuhai, Guangdong, China; ^4^ Department of Interventional Therapy, Sichuan Cancer Hospital and Institute, Sichuan Cancer Center, Chengdu, Sichuan, China; ^5^ College of Education, Jinan University, Zhuhai, Guangdong, China

**Keywords:** cerebral ischemia-reperfusion injury, neuroinflammation, ursolic acid, microglia, pyroptosis

## Abstract

**Introduction:**

Neuroinflammation is a pivotal factor in the pathophysiology process of ischemic stroke. Undue inflammatory responses are the main cause of neuronal death and infarct enlargement after reperfusion, but there is currently no effective clinical treatment method. Pyroptosis plays an important role in post-stroke neuroinflammation. Inhibiting pyroptosis may be a potential method for treating ischemic stroke. Ursolic acid (UA) is a natural antioxidant with an antipyroptotic effect, but the mechanism of UA in cerebral ischemia-reperfusion injury remains unknown.

**Methods:**

We evaluated UA’s neuroprotective effects in a transient middle cerebral artery occlusion (tMCAO) mouse model via oral administration. TTC staining was carried out to measure infarct volume. Neuronal damage was assessed through TUNEL and FJC staining. Open field test and novel object recognition test were conducted to evaluate anxiety-like behavior and hippocampal-related memory. Double-immunofluorescence was conducted to detect pyroptotic microglia. *In vitro*, BV-2 microglial cells subjected to oxygen-glucose deprivation/reoxygenation (OGD/R) were treated with UA. Cell viability was measured utilizing CCK-8 assay. RT-qPCR was used to measure NLRP3, IL-1β, and IL-18 mRNA levels. ELISA was utilized to measure IL-1β and IL-18 concentration. PMA was used as an agonist in rescue experiment. Immunostaining was used to observe nuclear/cytoplasmic distribution of NF-κB. Western blot was used to evaluate the protein expression of pyroptosis markers.

**Results:**

UA significantly reduced infarct volume, alleviated neuronal damage, and improved cognitive and functional recovery in tMCAO mice. Additionally, UA downregulated the density of NLRP3, Caspase-1, and GSDMD positive microglia and the production of IL-1β and IL-18 in the ischemic penumbra of tMCAO mice. These effects were replicated in OGD/R-challenged BV-2 cells. Mechanistically, UA suppressed NF-κB activation, and PMA treatment reversed its therapeutic benefits in both models.

**Discussion:**

Our findings demonstrate that UA attenuates microglial pyroptosis by inhibiting NF-κB signaling, thereby reducing neuroinflammation and ischemic brain injury. This study highlights UA’s potential as a therapeutic agent for ischemic stroke.

## 1 Introduction

Ischemic stroke is a major cerebrovascular disease resulting from an acute interruption of the blood flow reaching parts of the brain due to intracranial artery embolism or thrombosis. Public health is seriously compromised due to its high disability and mortality rates (Campbell and Khatri, 2020; Tu et al., 2023). Currently, the clinical treatment method is thrombolysis or thrombectomy within a short time window, which does not address postischemic inflammatory response (Saini et al., 2021). Microglial activation is a critical factor in the inflammatory response seen in cerebral ischemia/reperfusion injury (CIRI). Moreover, undue neuroinflammation could result in neuron death and neurological impairment after CIRI (Ma et al., 2017). Therefore, new strategies targeting post-stroke neuroinflammation are urgently needed.

Pyroptosis is a highly pro-inflammatory cell death mediated by the inflammasome (Bergsbaken et al., 2009). Up to now, various inflammasomes have been studied, and the nucleotide-binding oligomerization domain-like receptor pyrin domain containing 3 (NLRP3) inflammasome is the best-characterized one among all the inflammasome families (Schroder and Tschopp, 2010). When pathogen-associated molecular patterns (PAMPs) or damage-associated molecular patterns (DAMPs) are recognized and detected by pattern recognition receptors (PRRs), the downstream NF-κB pathway is triggered, resulting in upregulated transcription of NLRP3, inflammatory cytokines pro-interleukin (IL)-1β, and pro-IL-18 (Peng et al., 2020). Thereafter, the inflammasome-adaptor protein ASC assembles to bind with NLRP3 and then acts on pro-caspase-1, resulting in its self-cleavage. Mature caspase-1 can promote the maturation of pro-IL-1β and pro-IL-18 (Davis et al., 2011). Moreover, mature caspase-1 cleaves gasdermin D (GSDMD) into an active N-terminal fragment. These active fragments translocate from the cytoplasm to the cell membrane and induce membrane perforation, resulting in the leakage of proinflammatory cytokine and exacerbating the inflammatory cascades (Shi et al., 2015). Mounting evidence indicates that NLRP3-mediated microglia pyroptosis is essential to neuroinflammation following CIRI (Barrington et al., 2017; Franke et al., 2021). For example, Panax ginseng and Angelica sinensis administration diminished brain infarction volume and improved neurological outcomes by inhibiting NLRP3 inflammasome activation and microglia pyroptosis in MCAO rats (Hu et al., 2020). It has been shown that the stimulator of interferon genes (STING) activated the NLRP3 inflammasome pathway and that STING knockout could inhibit microglia pyroptosis and alleviate ischemic brain injury (Li et al., 2023). These findings indicate that suppressing NLRP3 inflammasome-mediated microglia pyroptosis is potentially valuable for treating ischemic stroke.

Ursolic acid (UA) is a natural compound extracted from traditional medicinal herbs and foods (Hill and Connolly, 2020). It has been proved that UA exerts neuroprotective effects in animal models of different brain disorders (Ramos-Hryb et al., 2017; Chen et al., 2023). For example, UA suppresses microglia pyroptosis to attenuate neuroinflammation in experimental cerebral hemorrhage (Lei et al., 2023). It has been indicated that UA can regulate the PDCD4/PI3K/AKT pathway through miR-141 and promote functional recovery in a traumatic brain injury mice model (Zhang et al., 2023). Previous studies reported that UA suppressed abnormal protein accumulation in hippocampi and delayed Parkinson’s disease progression in the early stage (Bang et al., 2023). However, whether UA has efficacy in suppressing NLRP3 inflammasome-mediated microglia pyroptosis in ischemic stroke is still unclear. The objective of this study is to demonstrate the therapeutic impact of UA in CIRI and to investigate whether the molecule mechanism of UA is associated with NF-κB/NLRP3-mediated microglia pyroptosis and neuroinflammation in ischemic stroke.

## 2 Materials and methods

### 2.1 Mice

Adult male C57BL/6 mice, 8–10 weeks old (22–28 g), were purchased from Guangdong Medical Laboratory Animal Center. Animal experiments were conducted and followed by the Ethical Guidelines on Laboratory Animal Welfare approved by the Ethical Review Committee of the Fifth Affiliated Hospital of Sun Yat-Sen University (2023112001–00440, Guangdong, China). All mice were housed in an environment with a standardized light-dark cycle and had *ad libitum* access to food and water. Mice were randomly divided into groups; the experimental design and animal grouping were presented in Supplementary Figure 1A; the mortality of mice was shown in Supplementary Table 1.

### 2.2 Transient middle cerebral artery occlusion (tMCAO) model establishment

MCAO surgery was conducted by obstructing the MCA using a nylon monofilament. Briefly, mice were anesthetized with 2% isoflurane. A midline cervical incision was made, and the right common carotid artery (CCA) was exposed. The external carotid artery (ECA) was ligated, and the filament was inserted through the CCA and the internal carotid artery (ICA). Then, the filament was advanced into the MCA to block blood flow from the contralateral hemisphere. After 45 min of ischemia, the filament was withdrawn to allow reperfusion. Sham-operated mice were anesthetized and accepted the identical operation aside from the occlusion.

### 2.3 Cell culture, oxygen-glucose deprivation/reoxygenation (OGD/R) model establishment

BV-2 microglia cells were cultured with Dulbecco’s modified Eagle medium (DMEM) containing 10% fetal bovine serum. Cells were cultured in an incubator in the environment of 5% CO_2_ at 37°C. OGD/R was conducted in accordance with previous methods. In brief, cell supernatants were replaced with glucose- and serum-free DMEM, and cells were transferred to an anaerobic chamber (Binder, Germany). After 2 h of OGD modelling, the medium was replaced by DMEM, and BV-2 cells were transferred to an aerobic chamber for reoxygenation. Control BV-2 cells were grown in a normal aerobic chamber for the same periods.

### 2.4 Drug administration

UA (10, 20 mg/kg, Selleck, United States) and MCC950(10 mg/kg, Selleck, United States) were dissolved in corn oil containing 1% DMSO. UA, MCC950, or corn oil were given intragastrically at 45 min following MCAO modelling. BV-2 cells were cultured with 4 µM UA to suppress pyroptosis. MCC950 (10 μM, Selleck, United States) was used as a positive control to inhibit NLRP3 activation. The rescue experiment used PMA (1 μM, Selleck, United States) as a potent agonist for NF-κB signal pathways. Cells were collected 24 h after drug administration for subsequent analyses.

### 2.5 Cell viability

The proliferative activities of BV-2 cells were measured utilizing the Cell Counting Kit-8 (CCK8) assay (Beyotime Biotechnology, China). BV-2 cells, with or without OGD challenge, were seeded in 96-well microplates, cultured, and stimulated with different dosages (1, 2, 4, 8, and 16 µM) of UA for 24 h. A microplate reader was utilized to determine the absorbance at 450 nm.

### 2.6 Neurobehavioral test

#### 2.6.1 Neurological function assessment

Neurological deficits were evaluated by Zea-Longa scoring criteria. Briefly, mice without apparent neurological deficits obtained 0, mice with signs of forelimb flexion obtained 1, mice with the body tilted to one side when crawling forward obtained 2, mice who fell to one side and crawled in circles obtained 3, and mice with comatose or moribund state obtained 4.

#### 2.6.2 Open field test (OFT)

The OFT was utilized to assess anxiety-like behavior. Briefly, mice were acclimated to the open-field apparatus for 10 min on two successive days before testing. Then, mice were placed into the center of the apparatus for 5 min. The mice track was recorded using a digital video-tracking system (Nanjing Calvin Biotechnology Co., Ltd., China).

#### 2.6.3 Novel objects recognition (NOR) test

The NOR test was carried out to evaluate hippocampal-related memory. Two familiar objects were placed diagonally at the open-field apparatus during the training task. Mice were allowed to explore the objects for 5 min freely. A novel object was replaced 1 h after one of the familiar objects, and mice were again placed into the apparatus to freely explore the objects for 5 min. The time spent exploring familiar and novel objects was recorded, and a preference index was calculated to assess hippocampal-related memory.

### 2.7 Measurements of infarct volume

2,3,5-Triphenyl-2H-tetrazolium chloride (TTC) staining was performed to distinguish the infarction area. At 7 days after reperfusion, mice were anesthetized and sacrificed. The brains were rapidly removed and frozen at −80°C for 8–10 min. After that, brains were coronally sliced into 2 mm thick sections and incubated in 2% TTC solution for 20 min at 37°C in the dark. After that, the brain sections were photographed, and the infarct volume of each slice was evaluated utilizing ImageJ software (NIH, United States).

### 2.8 Immunofluorescence

Mice were anesthetized and sacrificed. Precooled PBS and 4% PFA were used for perfusion and fixation, respectively. Brains were removed and successively incubated with 4% PFA, 20% sucrose, and 30% sucrose at 4°C overnight. Samples were embedded and cut into 20 μm frozen coronal sections. After a PBS wash, slices were dried at 60°C for 1 h and were blocked with 10% goat serum and 0.5% Triton X-100 for 1 h. Then, the sections were incubated with primary antibodies at 4°C overnight. After three PBS washes, slides were incubated with fluorochrome-labelled secondary antibodies for 1 h in the dark. Finally, slices were counterstained with DAPI for 5 min and sealed with mounting medium. The antibody information is provided in Supplementary Table 2.

BV-2 cells were cultured on confocal dishes. After OGD modelling and drug administration, cells were washed with PBS and fixated with 4% PFA. Then, cells were blocked with 10% goat serum and 0.1% Triton X-100 for 1 h. The subsequent protocols are the same as mentioned. Immunofluorescence images were taken utilizing a confocal microscope (Zeiss, Germany) or an upright microscope (Olympus, Japan). Positive microglia or fluorescence intensity was measured using ImageJ software.

### 2.9 TdT-mediated dUTP Nick-End labeling (TUNEL) and Fluoro-Jade C (FJC) staining

Apoptosis was detected with the One Step TUNEL Assay Kit (Servicebio, China). Briefly, frozen brain sections were incubated with Proteinase K for 20 min. After a PBS wash, sections were incubated with the TUNEL reaction mixture for 1 h at 37°C in the dark, with subsequent counterstaining with DAPI.

FJC staining was conducted to distinguish degenerated neurons (Biosensis, United States). Briefly, frozen brain sections were treated with NaOH/ethanol and potassium per-manganate for rehydration and penetration. After two ddH_2_O washes, sections were incubated with FJC solution for 10 min in the dark, with subsequent counterstaining with DAPI. The stained slides were examined under an upright microscope and counted positive cells.

### 2.10 Quantitative real-time PCR (qPCR)

mRNA was obtained from BV-2 cells or ischemic cortex samples using a Fast Pure Cell/Tissue Total RNA Isolation Kit (Vazyme Biotech, China). Reverse transcription and quantitative real-time PCR were performed on a thermal cycler (Bio-Rad, United States) utilizing the HiScript^®^ III RT SuperMix for qPCR (+gDNA wiper) reagent kit (Vazyme Biotech, China) and the ChamQ Universal SYBR qPCR kits (Vazyme Biotech, China) following the manufacturer’s instructions. The primer sequences are listed in Table 1.

**TABLE 1:** Primers information table.

Gene	Forward primer (5′ to 3′)	Reverse primer (5′ to 3′)
NLRP3	GAT​CAA​CAG​GCG​AGA​CCT​CTG	CCA​GCA​AAC​CCA​TCC​ACT​CTT
IL-1β	ACA​GGC​TCC​GAG​ATG​AAC​AAC	TCG​TTG​CTT​GGT​TCT​CCT​TGT
IL-18	GCT​GTG​ACC​CTC​TCT​GTG​AAG	TGT​CCT​GGA​ACA​CGT​TTC​TGA
β-actin	CAACGGCTCCGGCATGTG	AGT​CCT​TCT​GAC​CCA​TTC​CCA

### 2.11 Western blotting (WB)

BV-2 cells and ischemic brain tissues were lysed with RIPA Lysis Buffer (Beyotime, China). The proteins were segregated by SDS-PAGE and electro-transferred onto PVDF membranes (Millipore, United States). After a 1 h blocking, membranes were incubated with antibodies directed against NLRP3 (1:1000, #15101, Cell Signalling Technology, United States), cleaved-caspase-1 (1:1000, #89332, Cell Signalling Technology, United States), GSDMD-NT (1:1000, #10137, Cell Signalling Technology, United States), p-NF-κB (1:1000, ab76302, Abcam, United States), and β-actin (1:2000, ab8226, Abcam, United States) at 4°C overnight. Then, membranes were washed with TBST and incubated with horseradish peroxidase (HRP)-conjugated secondary antibodies. After three TBST washes, exposure was performed on a Molecular Imager Gel Doc XR System (Bio-Rad, United States). ImageJ was utilized to analyze the data.

### 2.12 ELISA

IL-1β and IL-18 concentration in ischemic cortex tissues or BV-2 cell supernatant were determined with ELISA kits (MIKX, China) directed by the manufacturer’s protocols.

### 2.13 Statistical analysis

GraphPad Prism 9.0 (GraphPad Software, United States) was utilized to analyze data. The data are presented as mean ± standard deviation (SD). One-way ANOVA followed by a post hoc Tukey’s test was applied to compare means. *P* < 0.05 was considered statistically significant.

## 3 Results

### 3.1 UA reduced cerebral infarction volume and neuronal damage in tMCAO mice

**FIGURE 1:**
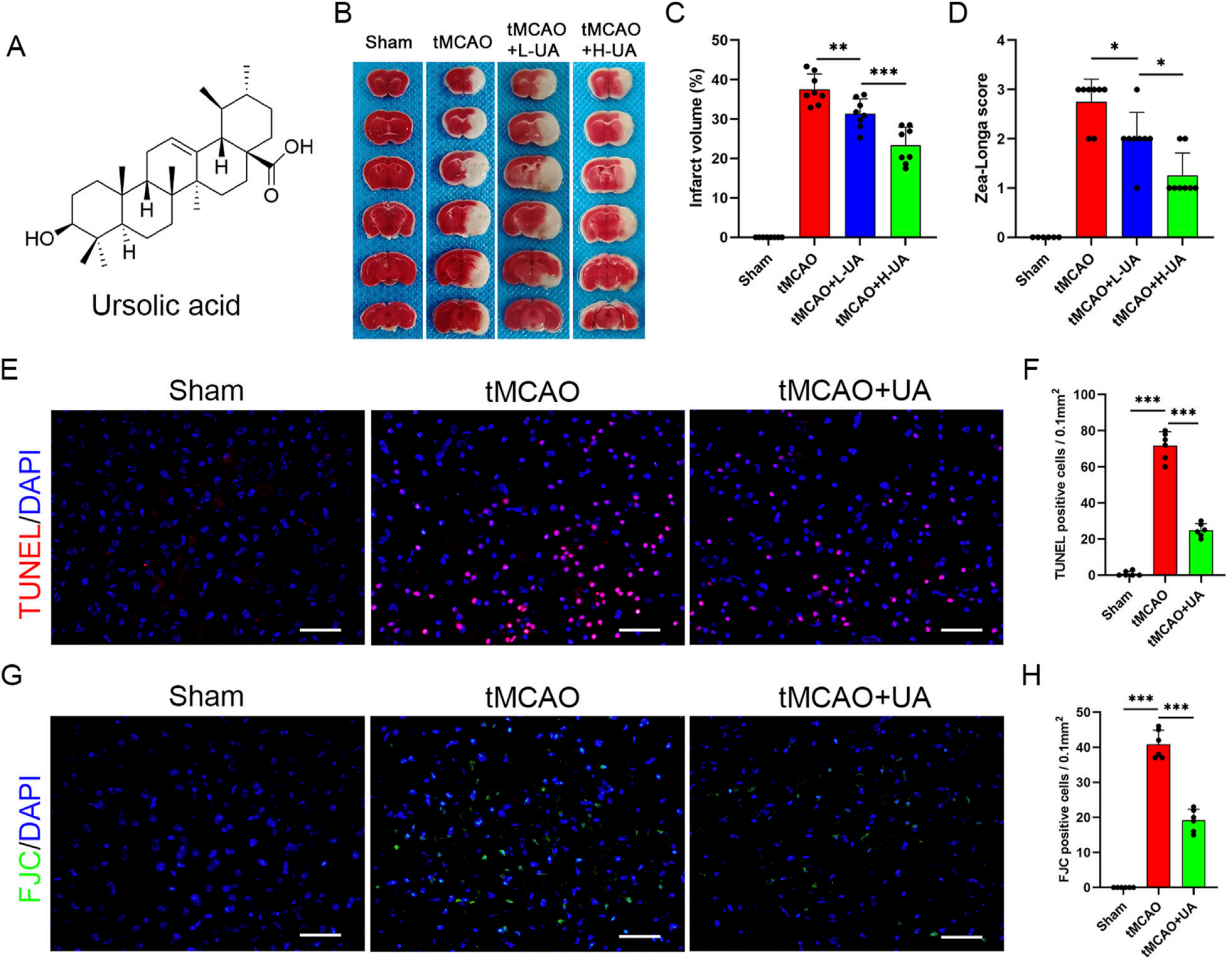
UA reduced infarct volume and neuronal damage in tMCAO mice. **(A)** Molecule structure of UA. **(B)** Representative TTC staining photographs in different groups after cerebral ischemia. **(C)** Measurement of infarct volume (n = 8). **(D)** Zea-Longa score was assessed in male mice 7 days after CIRI (n = 8). **(E–H)** TUNEL and FJC staining were performed to detect apoptosis and degenerative brain cells in tMCAO mice (n = 6). Scale bar: 50 μm *P < 0.05, **P < 0.01, ***P < 0.001.

UA is a natural pentacyclic triterpenoid; the compound structure of UA is presented in Figure 1A. To verify its therapeutic impact, a tMCAO model was employed in mice, followed by the administration of UA through oral gavage for 7 days. TTC staining was subsequently utilized to measure the volume of cerebral infarction (Figure 1B). The results showed that UA administration effectively reduced infarct volume at day 7 post-MCAO. The high-dosage group (H-UA, 20 mg/kg) had a more significant effect than the low-dosage group (L-UA, 10 mg/kg) on reducing brain infarction (Figure 1C). Likewise, UA administration also decreased the Zea-Longa score dose-dependently at day 7 after MCAO (Figure 1D). Consequently, a 20 mg/kg dose was selected for the following experiments. These results unequivocally indicated that UA exerted neuroprotection after MCAO.

To further validate the neuroprotective effect of UA, TUNEL and FJC staining were conducted to detect neuronal damage in the peri-infarct penumbra in mice (Figures 1E,G). The results revealed the elevated levels of TUNEL-positive cells in tMCAO mice, which dramatically decreased following UA treatment (Figure 1F). Additionally, FJC staining evidenced a considerable decrease in the density of degenerating neurons in mice treated with UA, in stark contrast to tMCAO + vehicle mice (Figure 1H). These findings collectively suggested that UA exhibited a neuroprotective effect following MCAO, underscoring its potential as a therapeutic agent in mitigating neuronal damage.

### 3.2 UA improved cognitive deficits and promoted functional recovery after ischemic stroke

**FIGURE 2:**
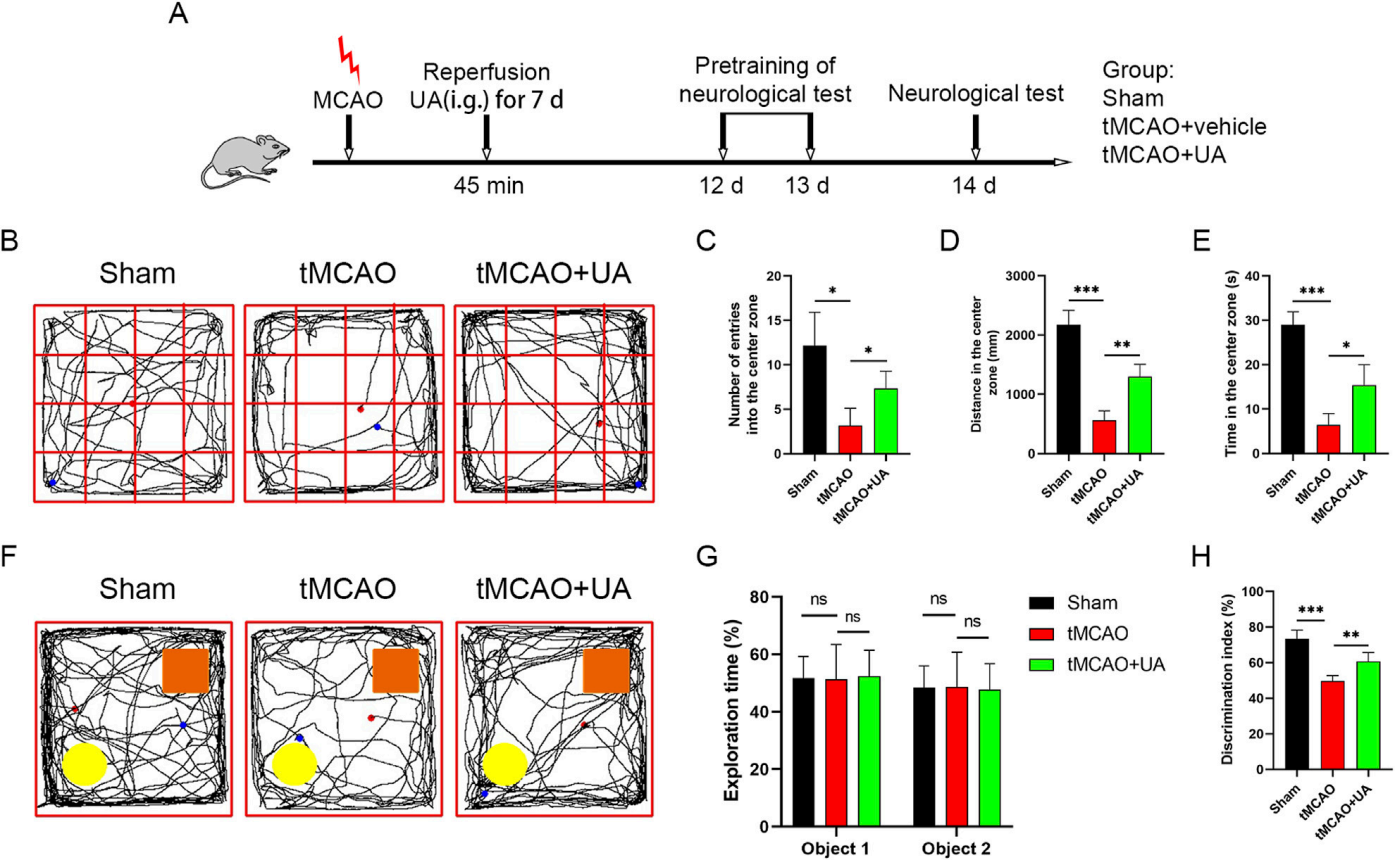
UA improved cognitive function and promoted functional recovery in tMCAO mice. **(A)** Experimental design and animal groups. **(B)** In the OFT, sensorimotor impairment and anxious behavior were tested 14 days after tMCAO. **(C–E)** Quantitative analysis of entries into the center zone, distance in the center zone, and time spent there. **(F)** In the NOR test, cognitive function was evaluated 14 days after tMCAO. Orange rectangle: familiar object. Yellow circle: novel object. **(G,H)** Quantitative analysis of exploration time and discrimination index. n = 6 per group. *P < 0.05, **P < 0.01, ***P < 0.001.

Next, to elucidate the long-term therapeutic impact of UA, we conducted an open field test (OFT) and novel object recognition (NOR) test at 14 days post-MCAO; the experimental design was presented in Figure 2A. Cognitive function was assessed by OFT (Figure 2B). As shown in Figures 2C–E, in contrast to sham-operated mice, entries into the center zone, distance in the center zone, and time in the center zone were profoundly diminished in tMCAO mice, suggesting that MCAO surgery caused cognitive impairments such as anxiety and depression. However, UA-treated MCAO mice exhibited shorter distances traveled and time spent in the center zone, suggesting that UA facilitated recovery of cognitive function. Subsequently. A NOR test was then conducted to detect cognitive improvement (Figure 2F). During the training task, mice explored both identical objects with no preference (Figure 2G). In contrast, during the recognition task, mice treated with UA displayed a preference for the novel object over tMCAO + vehicle mice, implying that they retained intact memory of familiar objects (Figure 2H). Collectively, these results revealed that administration of UA significantly improved cognitive deficits after ischemic stroke.

### 3.3 UA alleviated microglia pyroptosis induced by ischemic insult

**FIGURE 3:**
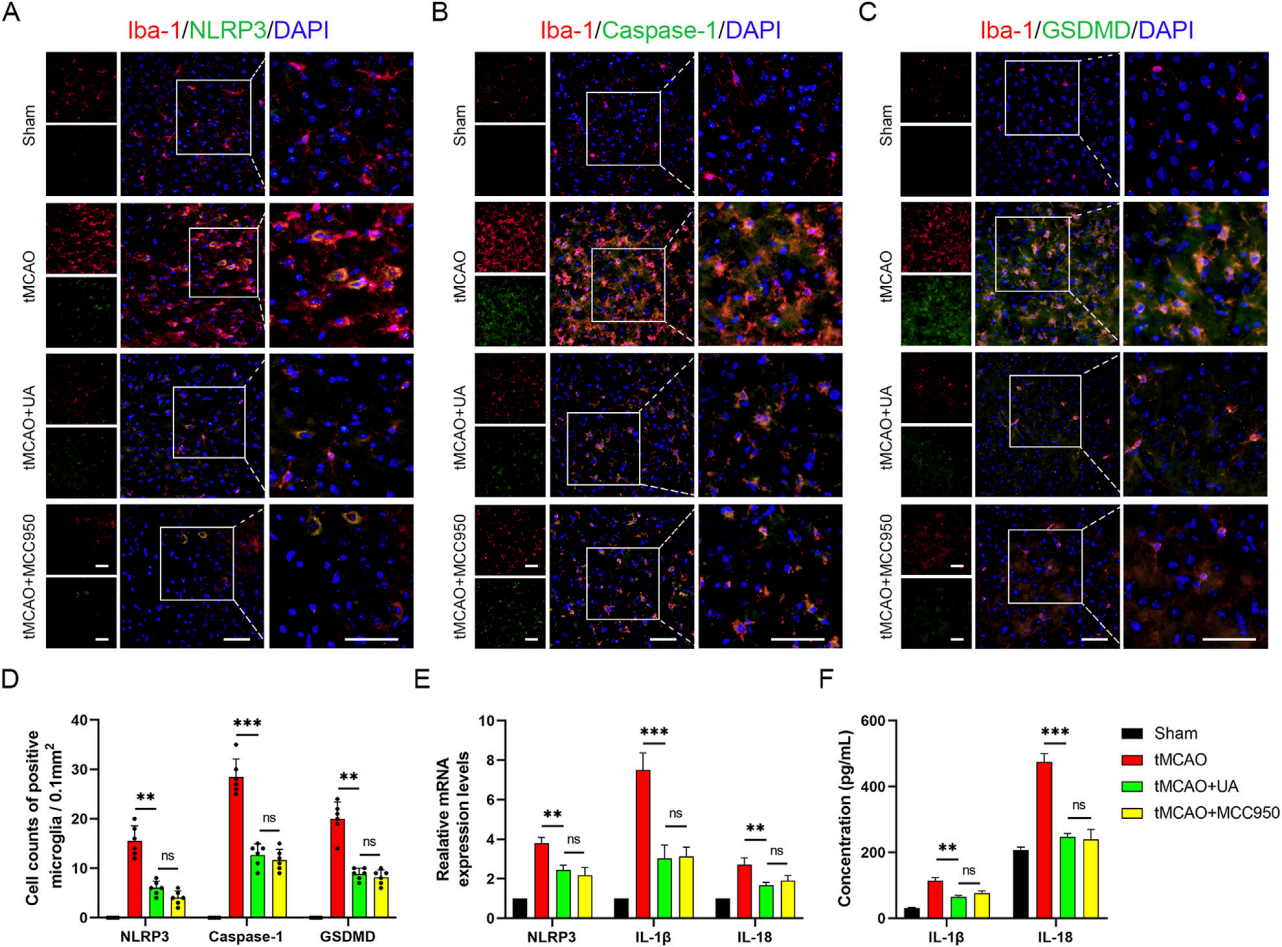
UA inhibited NLRP3-mediated microglia pyroptosis in tMCAO mice. **(A–C)** Double immunofluorescence of Iba-1 and NLRP3, caspase-1, GSDMD in the pe-ri-infarct penumbra 7 days after reperfusion (n = 6). Scale bar: 50 µm. **(D)** Cell counts of double immunostaining. **(E)** NLRP3, IL-1β, and IL-18 mRNA levels in the cerebral ischemic area (n = 3). **(F)** IL-1β and IL-18 concentrations in brain tissues were measured by ELISA (n = 5). *P < 0.05, **P < 0.01, ***P < 0.001.

Pyroptosis plays a vital role in post-stroke neuroinflammation. To explore the potential association between UA and pyroptosis-mediated neuroprotection, we conducted double immunofluorescence in mice brain sections. As depicted in Figures 3A–C, microglia were labeled by anti-Iba-1 antibody, and pyroptotic cells were labeled by NLRP3, caspase-1, and GSDMD. The results unveiled an augmented presence of NLRP3, caspase-1, and GSDMD-positive microglia in the ischemic penumbra after reperfusion injuries; however, this effect was counteracted by the administration of UA or MCC950 (10 mg/kg) (Figure 3D). MCC950 is an NLRP3 inflammasome inhibitor used as a positive control. qPCR data analysis exhibited that IL-1β, IL-18, and NLRP3 transcription levels were also profoundly promoted in tMCAO mice, but then were downregulated after UA or MCC950 administration (Figure 3E). Concurrently, ELISA results showed that the concentration of IL-1β and IL-18 in ischemic penumbra was inhibited by UA or MCC950 (Figure 3F). The combined results suggested that CIRI activated the NLRP3/Caspase-1/GSDMD-mediated microglia pyroptosis, leading to the unregulated expression of cytokines in mice. However, administration of UA effectively suppresses these patterns, indicating its potential as a neuroprotective agent by inhibiting microglia pyroptosis.

### 3.4 UA promoted OGD/R-challenged BV-2 cells survival by inhibiting NLRP3-mediated pyroptosis

**FIGURE 4:**
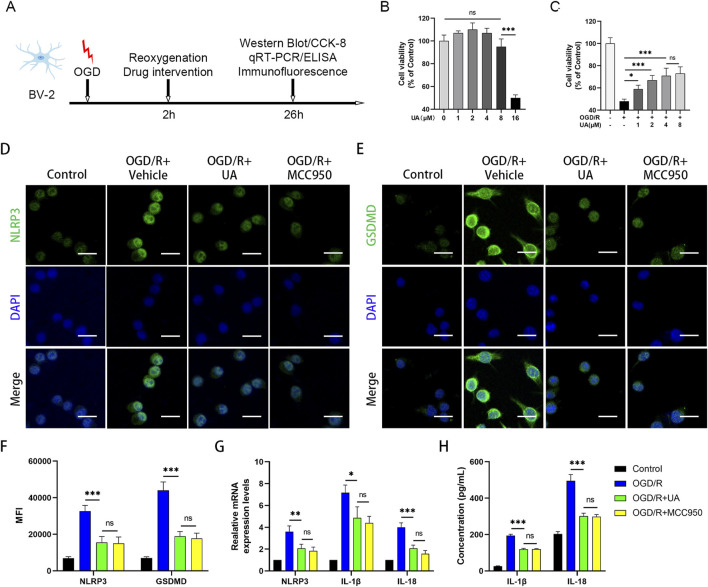
UA alleviated NLRP3-microglia pyroptosis in OGD/R-challenged BV-2 cells. **(A)** Experimental design. **(B,C)** Cell viability of UA-treated BV-2 cells in normal and OGD conditions (n = 5). **(D,E)** Immunostaining of NLRP3 and GSDMD in BV-2 cells. Scale bar: 25 µm. **(F)** Mean fluorescence intensity of pyroptosis-related proteins (n = 3). **(G)** mRNA levels of NLRP3 and cytokines in BV-2 cells (n = 3). **(H)** Cytokines production in BV-2 cell supernatants were measured by ELISA (n = 5). *P < 0.05, **P < 0.01, ***P < 0.001.

Encouraged by the results obtained from the *in vivo* experiment, we next sought to validate the anti-pyroptotic effect of UA in BV-2 microglia cells. Firstly, we established the OGD/R model as previously described. To determine the reperfusion duration, we performed PCR and Western blot experiments to observe the expression of NLRP3 at different time points after OGD. The results demonstrated that NLRP3 mRNA and protein expression was markedly elevated at 24 h post reoxygenation compared to earlier time points (Supplementary Figures 2A‐C). Therefore, 24 h observation window was selected in subsequent experiments; the experimental design was presented in Figure 4A. Cell viability of BV-2 cells was evaluated using CCK-8 kits. Subsequently, BV-2 cells were cultured with varying concentrations of UA (0, 1, 2, 4, 8, and 16 μM) for a duration of 24 h. Notably, it was observed that the half-maximal inhibitory concentration (IC_50_) of UA in BV-2 cells was 16 μM (Figure 4B). In addition, cell viability was reduced to 48% after the OGD/R challenge, while UA could dose-dependently increase cell viability after reoxygenation (Figure 4C). To determine the appropriate drug concentration, we conducted qPCR experiments. 0, 2, 4 and 8 μM UA were administered to BV2 cells after OGD/R modelling, and the mRNA expressions of pyroptosis markers were measured. As shown in Supplementary Figure 2D, the quantitative analysis results showed that the mRNA levels of NLRP3, IL-1β, and IL‐18 in the UA‐treated group were significantly lower compared to the vehicle group. Notably, 4 μM and 8 μM groups exhibited stronger effects than the 2 μM group, while no significant difference between the 4 μM and 8 μM groups. These results suggested 4 μM UA as an effective and safe dose for mechanistic studies. Therefore, 4 μM UA was used in subsequent in vitro experiments. Immunofluorescence analysis targeting NLRP3 and GSDMD proteins within BV-2 cells was conducted through immunostaining techniques. As depicted in Figures 4D,E, the average fluorescence intensity of NLRP3 and GSDMD was profoundly enhanced after OGD/R. However, this trend was reversed upon administration of UA or MCC950 (Figure 4F). qPCR analysis confirmed the upregulation of cytokines and NLRP3 transcription levels in the OGD/R group but subsequently downregulated after treatment with UA or MCC950 (Figure 4G). Concurrently, ELISA results demonstrated that the production of cytokines induced by OGD/R in the supernatant of BV-2 cells was suppressed by UA and MCC950 (Figure 4H). Altogether, these findings suggested that UA potently suppressed microglia pyroptosis *in vitro*.

### 3.5 UA suppressed OGD/R-induced NLRP3-mediated pyroptosis by preventing NF-κB p65 phosphorylation and translocation in OGD/R-challenged BV-2 cells

**FIGURE 5:**
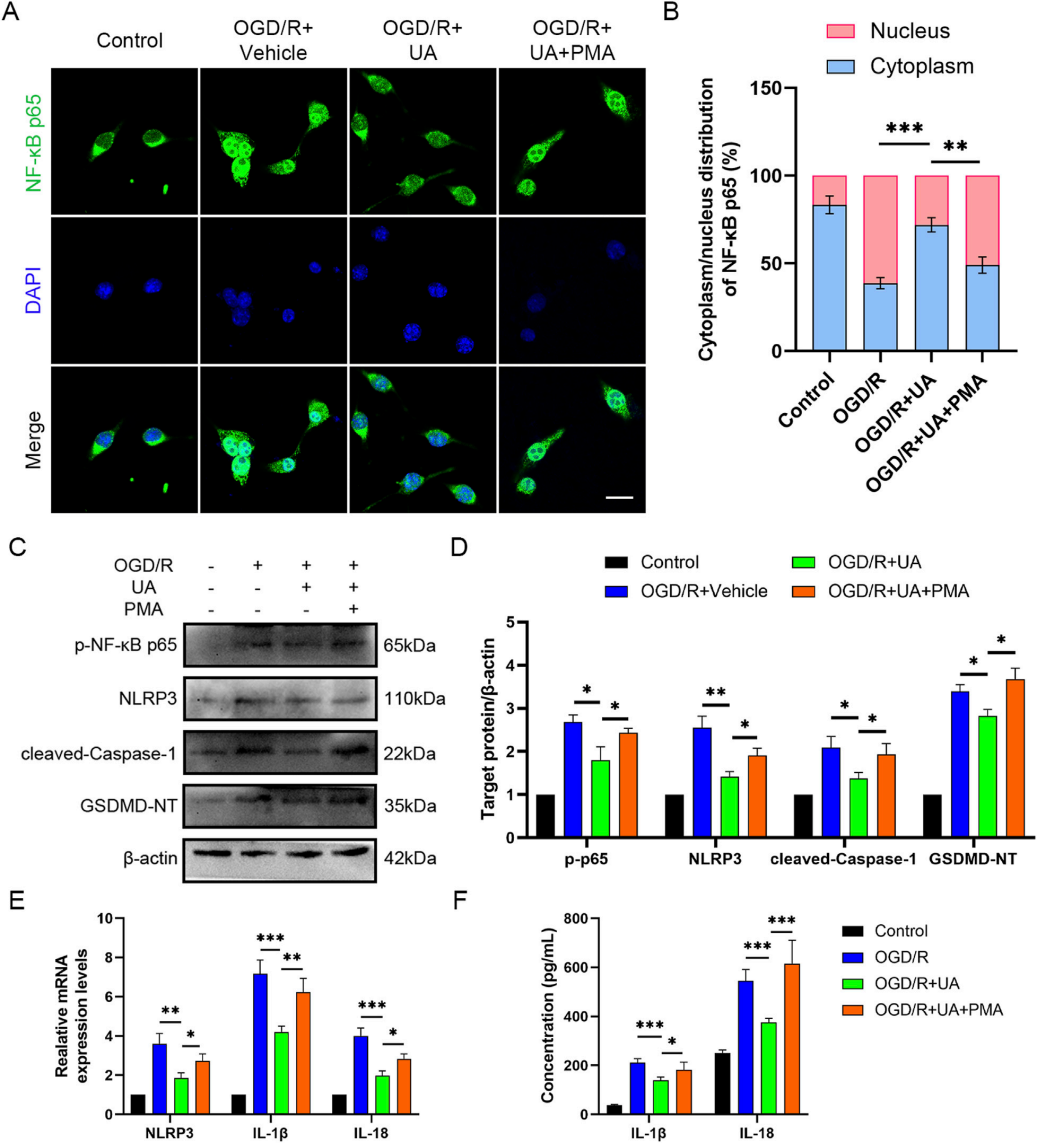
UA suppressed microglial pyroptosis by preventing NF-κB p65 phosphorylation and translocation *in vitro*. **(A)** Immunostaining of NF-κB p65 in BV-2 cells 24 h following OGD/R challenge. Scale bar: 25 µm. **(B)** Cytoplasm/nucleus distribution analysis. **(C,D)** p-NF-κB, NLRP3, Cleaved-caspase-1, and GSDMD-NT protein levels in different groups (n = 3). **(E)** NLRP and cytokines mRNA levels in different groups (n = 3). **(F)** Cytokines production in BV-2 cell supernatants (n = 5). *P < 0.05, **P < 0.01, ***P < 0.001.

Furthermore, to investigate the mechanism by which UA regulated pyroptosis in OGD/R-challenged BV-2 cells, we focused on the NF-κB pathways, which play a crucial role in ischemic stroke (Xu et al., 2024). Our aim was to explore whether NF-κB signalling can influence the therapeutic effect of UA. Therefore, a potent NF-κB agonist PMA was used to activate NF-κB signalling. The immunostaining results showed that UA suppressed p65 nuclear translocation in OGD/R-challenged BV-2 cells; however, this trend was blocked by PMA administration (Figures 5A,B). Moreover, in contrast to the OGD/R + UA group, the OGD/R + UA + PMA group exhibited elevated protein levels of p-p65, NLRP3, cleaved-Caspase-1, and GSDMD 24 h after reoxygenation (Figures 5C,D). qPCR results demonstrated that compared with the OGD/R group, IL-1β, IL-18, and NLRP3 transcription levels were downregulated after UA administration, but PMA abolished the therapeutic effect of UA (Figure 5E). Likewise, ELISA assay demonstrated that OGD/R induced IL-1β and IL-18 production in BV-2 cells supernatant was suppressed by UA. Nevertheless, the PMA administration reversed this trend (Figure 5F). In sum, the data presented supported the hypothesis that UA mitigated NLRP3-mediated pyroptosis induced by OGD/R at least partly through the prevention of NF-κB p65 phosphorylation and translocation in OGD/R-challenged BV-2 cells.

### 3.6 UA inhibited CIRI-induced NLRP3-mediated microglia pyroptosis by preventing NF-κB p65 activation in MCAO mice

**FIGURE 6:**
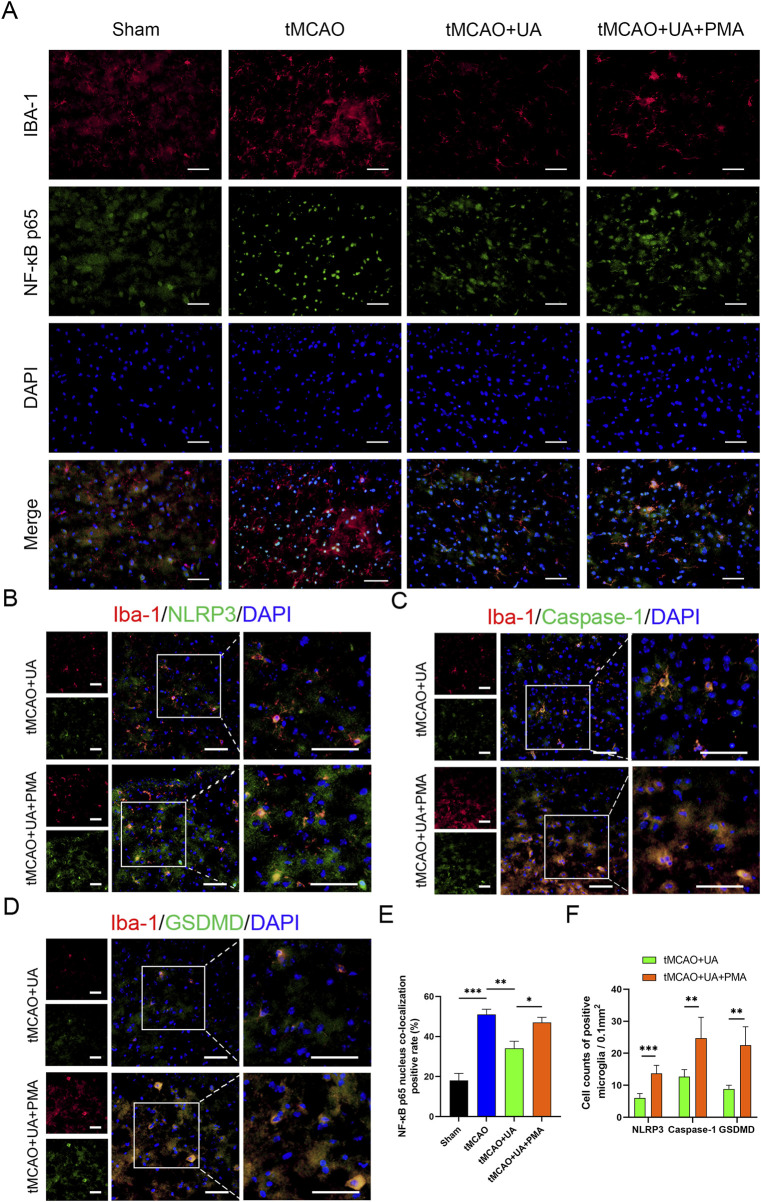
UA suppressed microglial pyroptosis by preventing NF-κB p65 translocation *in vivo*. **(A)** Immunostaining of NF-κB p65 in ischemic brain tissue of MCAO mice (n = 6). Scale bar: 50 µm. **(B–D)** Double immunofluorescence of Iba-1 and NLRP3, caspase-1, GSDMD in the peri-infarct penumbra (n = 6). Scale bar: 50 µm. **(E)** Percentage of NF-κB nucleus colocalization positive cells. **(F)** Cell counts of positive microglia. *P < 0.05, **P < 0.01, ***P < 0.001.

To further validate the mechanistic axis in the MCAO mice brain, we conducted double immunofluorescence staining for NF-κB p65 and Iba-1. The merged images exhibited yellow fluorescence, signifying the co-localization of NF-κB p65 staining (green) with Iba1 (red) (Figure 6A). From Figure 6A, it can be observed that in the sham group, NF-κB p65 was primarily localized in the cytoplasm, whereas the MCAO group exhibited significant nuclear translocation of NF-κB p65. Treatment with UA suppressed nuclear translocation, indicating that UA can inhibit NF-κB p65 activation. However, in the UA + PMA co-treatment group, this inhibitory effect was reversed (Figure 6E).

Additionally, to observe the effect of PMA on pyroptotic molecules, we extended the experiment from Figure 3A and added an MCAO + UA + PMA group (Figures 6B–D). The results demonstrated that the UA + PMA co-treatment group exhibited a significant increase in NLRP3/Caspase-1/GSDMD-positive microglia compared to the UA-only group (Figure 6F), confirming that PMA counteracts UA’s anti-pyroptotic effect. These results further suggested that UA exert anti-pyroptotic effect by inhibiting NF-κB/NLRP3 pathway.

## 4 Discussion

**FIGURE 7:**
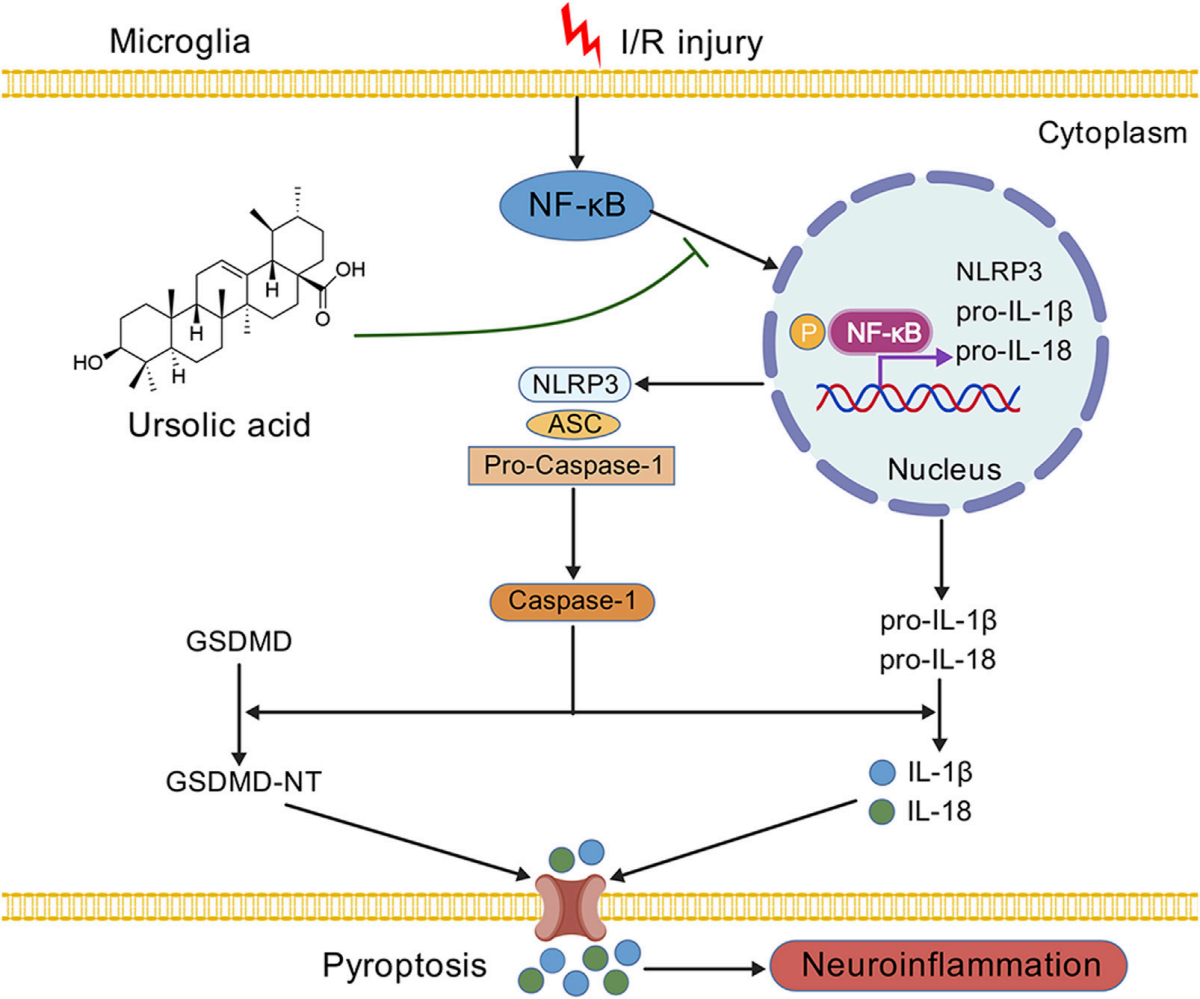
A schematic diagram displaying the underlying mechanism of UA in ischemic stroke. We demonstrated that the administration of UA may exert neuroprotection and ameliorate neuroinflammation after CIRI, possibly by inhibiting NF-κB activation and NLRP3 inflammasome-mediated microglia pyroptosis.

Ursolic acid (UA) is extensively utilized as an anti-inflammatory drug, and increasing evidence has proven that UA can exert neuroprotection in neurodegenerative and psychiatric diseases (Mirza et al., 2021; NAß and Efferth, 2021; Rai et al., 2019). Nevertheless, whether UA can inhibit microglia pyroptosis after ischemic stroke remains to be revealed. In this study, to explore the therapeutic impacts of UA following CIRI, a mice model of tMCAO and an *in vitro* ischemic stroke model of OGD/R-challenged BV2 cells were constructed. During animal experiments, we noticed that the infarct volume, neurological deficits, motor functions, and neuronal damage of MCAO mice were all significantly improved after the administration of UA, indicating that UA exerts neuroprotection in mice with ischemic stroke. In addition, we found that UA can suppress microglia pyroptosis as effectively as MCC950, a potent, selective NLRP3 inhibitor, by immunofluorescence, qPCR, Western blot, and ELISA. Moreover, we measured pro-inflammatory cytokine levels in the ischemic cortex of mice that underwent different interventions and found that UA can block the inflammatory response following ischemic stroke. In addition, to further unravel the mechanism of UA on microglia, we simulated the ischemic environment *in vitro* by constructing an OGD/R model in BV2 cells. The results suggested that UA adequately suppressed p65 nuclear translocation and phosphorylation, which subsequently downregulated the transcriptional expression of NLRP3, IL-1β, and IL-18. Besides, the mean immunofluorescence intensity of NLRP3 and GSDMD was suppressed by UA. IL-1β and IL-18 production also reduced after UA administration, suggesting that UA inhibited pyroptosis in BV-2 cells. However, the anti-pyroptotic effect of UA vanished after we administrated an NF-κB pathway agonist PMA to the system, indicating that the inhibitory effect of UA on inflammatory response may rely on blocking the NF-κB pathway (Figure 7).

Cerebral ischemia has contributed to mounting cases of death and disability worldwide (Saini et al., 2021). Currently, clinical treatments are focused on removing clots and building early reperfusion to rescue as many brain cells as possible (Macrez et al., 2011). Nevertheless, alleviating neurological deficits and promoting functional recovery after cerebral ischemia remains a major challenge on account of the nonregenerative nature of nerve cells. Thus, discovering new drugs to rescue neuronal cells from death has become an essential goal for promoting recovery following ischemic stroke.

Neuronal damage following CIRI generally derives from primary damage driven by brain edema and secondary damage driven by inflammation response (Shi et al., 2019). Since primary damage is inevitable, prevailing interventions for ischemic stroke largely concentrate on minimizing secondary damage after reperfusion. Neuroinflammation following cerebral ischemia is pivotal in driving secondary neuronal damage (Zhang et al., 2024). Microglia are responsible for immune surveillance as innate macrophages in the central nervous system, and microglia-mediated neuroinflammatory response plays an essential role in CIRI (Yong et al., 2019). After cerebral ischemia, microglia rapidly activate and polarize into the M1 phenotype and release pro-inflammatory cytokines, thereby exacerbating neuroinflammation (Jin et al., 2010). Thus, regulating the undue activation of microglia is crucial for the treatment of ischemic stroke.

Mounting evidence suggests that pyroptosis is one of the most significant programmed cell death modalities in microglia after cerebral stroke. It has been demonstrated that microglia pyroptosis is seen and contributes to neuroinflammation in mice with cerebral venous sinus thrombosis (Ding et al., 2022). Besides, TRIM29 interacts with NLRC4 inflammasome and attenuates apoptosis and pyroptosis of neurons and microglia cells in ischemic stroke (Deng et al., 2023). In addition, AIM2 inflammasome contributes to neuroinflammation and cognitive impairment in MCAO mice (Kim et al., 2020). Moreover, it has been illustrated that ischemic preconditioning exerts neuroprotective effects by suppressing NLRP3 inflammasome activation and microglia pyroptosis (Gao et al., 2023). Furthermore, HAMI 3379 is found to alleviate post-stroke depression as a CysLT (2)R antagonist by inhibiting NLRP3-mediated pyroptosis in gerbils (Zhou et al., 2022). Taken together, targeting inflammasome-mediated microglia pyroptosis may be a potent strategy for the treatment of CIRI.

In the current article, we preliminarily elucidated that UA inhibits microglia pyroptosis-mediated neuroinflammation after ischemic stroke, at least partly by inhibiting the NF-κB/NLRP3 pathway. Numerous factors and pathways may be involved in the therapeutic effects of UA, such as mediating microglia activation, polarization, and pyroptosis (Wang et al., 2023; Liu et al., 2024). More work is needed to focus on other relevant pathways that are likely related to the therapeutic effects of UA. It is difficult to distinguish the exact target proteins of UA. Future studies may utilize proteomics and transcriptomics techniques to predict and validate potential target spots through gene knockout mice and cell lines. The NF-κB/NLRP3/GSDMD pathway is a significant anti-pyroptotic and neuroprotective pathway after ischemic stroke. However, more target spots and mechanisms of UA under a hypoxic environment need to be further studied.

There are two main types of *in vitro* cell models that are used to simulate neuroinflammation. One is lipopolysaccharide-stimulated brain cells, which are more prevalent in the field of psychiatric and neurodegenerative diseases, including depression and Alzheimer’s disease. The other is oxygen deprivation/reoxygenation-challenged brain cells. Studies have been carried out to discover that similar pathological events such as mitochondrial damage and reactive oxygen species bust would occur after reoxygenation (Qin et al., 2022). Consequently, we chose the OGD/R model because it is an ideal model to investigate the pathological mechanism of ischemic stroke. Furthermore, we conducted preliminary investigations on BV2 cell polarization before and after OGD modeling (Supplementary Figure 3). The results indicated that M1‐polarized microglia predominated in the OGD group, whereas control group cells primarily maintained a resting state. However, we did not further explore the effect of UA in microglial polarization. The potential modulatory effects of UA on microglial polarization remain to be elucidated, representing an important direction for future studies.

This study has certain limitations. Firstly, BV-2 cells cannot fully replicate the microglia-mediated neuroinflammation after ischemic stroke. If needed, future studies may use primary microglia to conduct further investigation because the molecular properties and function of primary cells are more realistic and, thereby, more persuasive. Secondly, our *in vivo* rescue experiments were limited to immunofluorescence assay. Future studies should conduct *in vivo* rescue experiments at the molecular level to further validate the anti-pyroptotic mechanism of UA. Lastly, our focus was solely on investigating the effects of UA in microglia. Future studies should investigate whether UA can directly exert anti-inflammatory effects on neurons and other brain cells.

## 5 Conclusion

In summary, our findings suggest that UA exerts a neuroprotective effect by mitigating neuroinflammation and preventing secondary damage following ischemic stroke. This indicates the potential of UA as a promising therapeutic agent for CIRI. Furthermore, this study elucidated the intricate interplay between UA and the NF-κB pathway as well as NLRP3-mediated pyroptosis, thereby enhancing our understanding of the mechanisms underlying UA-mediated neuroprotection after ischemic stroke.

## Data Availability

The original contributions presented in the study are included in the article/Supplementary Material, further inquiries can be directed to the corresponding authors.
