# Supplementary material for: Ursolic acid ameliorates cerebral ischemia-reperfusion injury by inhibiting NF-κB/NLRP3-mediated microglia pyroptosis and neuroinflammation
Source: Front Pharmacol. 2025 Jul 11;16:1622131. doi: 10.3389/fphar.2025.1622131 (PMC12290296; doi:10.3389/fphar.2025.1622131)
Supplement: Supplementary file 1 [file Supplementaryfile1.docx]

**Supplementary Materials**


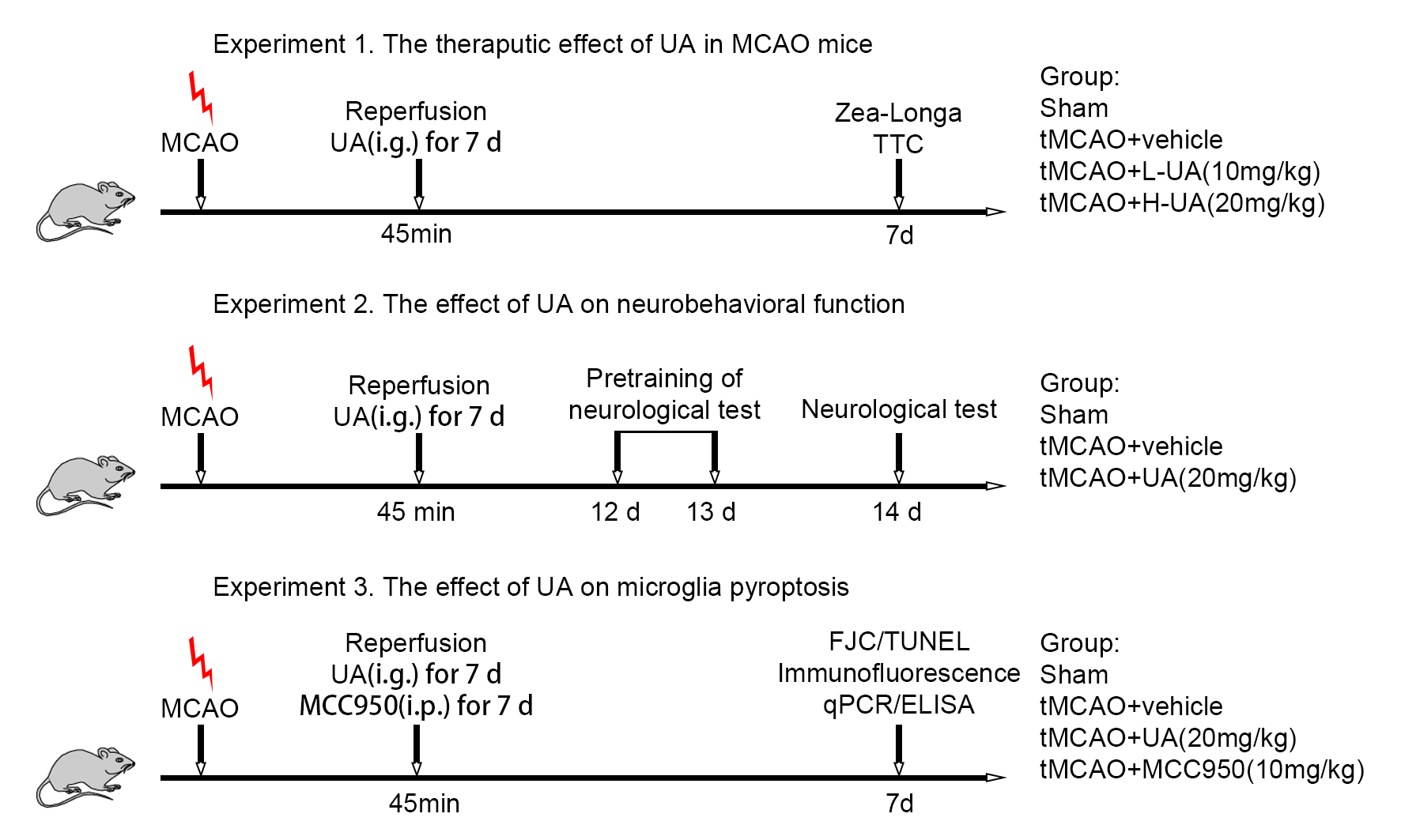


**Supplementary Figure. 1.** Experimental design of *in vivo* experiments. In Experiment 1, C57 mice were divided into 4 groups: sham operation, MCAO, MCAO+L-UA (10 mg/kg), and MCAO+H-UA (20 mg/kg), with 8 mice in each group, for Zea-Longa scoring and TTC staining experiments 7 days after reperfusion. In Experiment 2, the mice were divided into 3 groups: sham operation, MCAO, and MCAO+UA, with 6 mice in each group, for behavioral tests 14days after reperfusion. In Experiment 3, the mice were divided into 4 groups: sham operation, MCAO, MCAO+UA, and MCAO+MCC950, with 6 mice in each group, for brain tissue immunofluorescence staining experiments as well as qPCR/ELISA experiments 7 days after reperfusion.

**
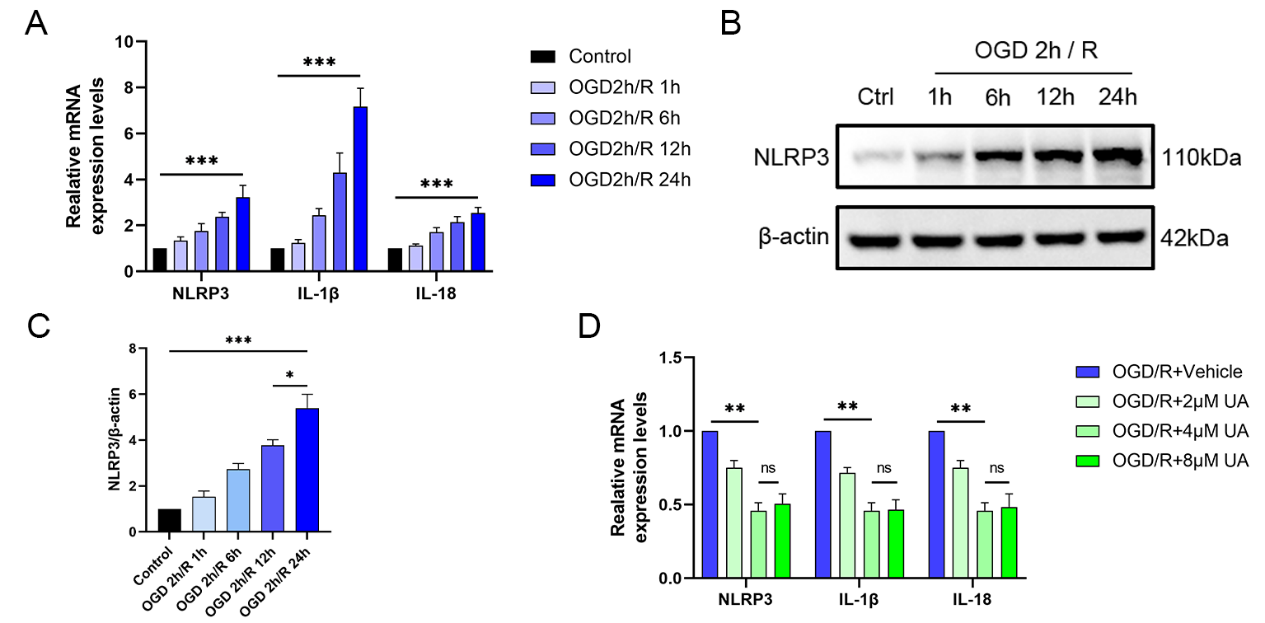
Supplementary Figure. 2.** Time points and drug dosage selection of in vitro experiments. (A) Quantitative analysis of pyroptosis markers at different time points after reoxygenation (n=3). (B) Western blotting to detect NLRP3 expression at different time points after reoxygenation. (C) Quantitative analysis of western blotting (n=3). (D) Quantitative analysis of pyroptosis markers with different concentrations of UA (n=3). *P<0.05, **P<0.01, ***P<0.001.

**
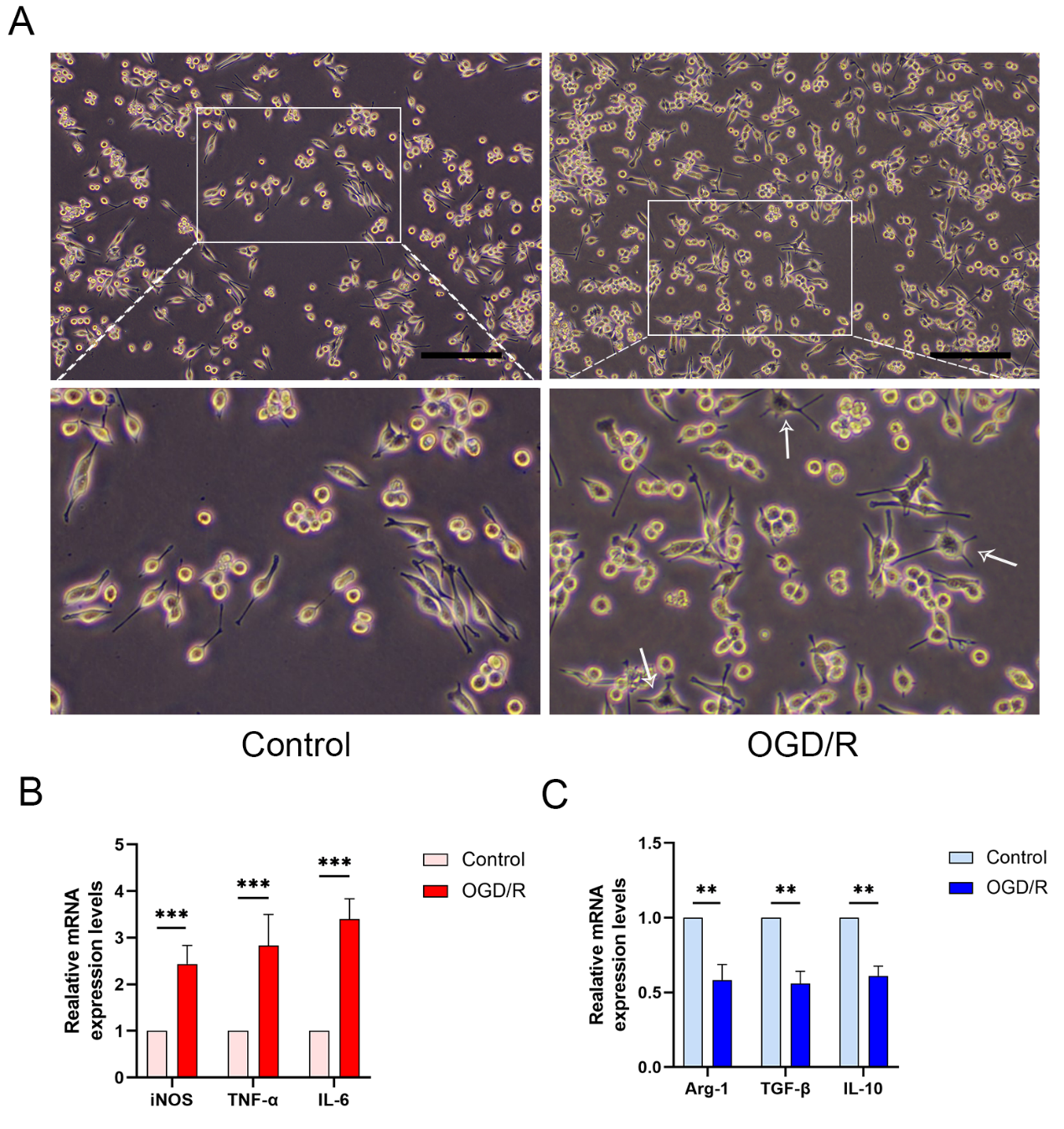
Supplementary Figure. 3.** Phenotypic identification of in vitro BV2 microglia cells. (A) Morphological changes in BV2 cells under bright field. (B) Quantitative analysis of mRNA levels of M1 markers (n=3). (C) Quantitative analysis of mRNA levels of M2 markers (n=3). Scale bar: 200 µm. **P<0.01, ***P<0.001.

**Table S1.** Animal usage and mortality of all the experimental groups.

| Group | Mortality | Excluded（reason） |
| --- | --- | --- |
| **Experiment 1** |  |  |
| Sham | 0% (0/8) | 0 |
| tMCAO+vehicle | 18.18% (2/11) | 1 (Cerebral hemorrhage) |
| tMCAO+L-UA (10mg/kg) | 10% (1/10) | 1 (No infarction) |
| tMCAO+H-UA (20mg/kg) | 11.11% (1/9) | 0 |
| **Experiment 2** |  |  |
| Sham | 0% (0/6) | 0 |
| tMCAO+vehicle | 22.22% (2/9) | 1 (Cerebral hemorrhage) |
| tMCAO+UA (20mg/kg) | 14.29% (1/7) | 0 |
| **Experiment 3** |  |  |
| Sham | 0% (0/6) | 0 |
| tMCAO+vehicle | 22.22% (2/9) | 1 (Poor eyesight during open field test) |
| tMCAO+UA (20mg/kg) | 14.29% (1/7) | 0 |
| tMCAO+MCC950 (10mg/kg) | 14.29% (1/7) |  |
| Total |  |  |
| Sham | 0% (0/20) | 0 |
| tMCAO | 15.94% (11/69) | 4 |

**Table S2.** Antibodies used in this study.

| Antibody | Manufacturer | Catalogue number | Dilution | Molecular weight (kDa) |
| --- | --- | --- | --- | --- |
| anti-NLRP3 | Cell Signaling Technology | #15101 | 1:1000 | 110 |
| anti-cleaved-Caspase-1 | Cell Signaling Technology | #89332 | 1:1000 | 22 |
| anti- cleaved-GSDMD | Cell Signaling Technology | #10137 | 1:1000 | 35 |
| anti-p-p65 | Abcam | ab76302 | 1:1000 | 65 |
| anti-β-actin | Abcam | ab8226 | 1:1000 | 42 |
| anti-Iba1 | Abcam | ab283319 | 1:100 | - |
| anti-NLRP3 | Novus | NBP2-12446 | 1:100 | - |
| anti-Caspase-1 | Novus | NBP1-45433 | 1:100 |  |
| anti-GSDMD | Affinity Biosciences | AF4012 | 1:100 | - |
| IgG H&L (Alexa Fluor® 488) | Abcam | ab150077 | 1:800 | - |
| IgG H&L (Alexa Fluor® 647) | Abcam | ab150115 | 1:800 | - |
| Dylight 594, Goat Anti-Mouse IgG | Abbkine | A23410 | 1:800 | - |
